# Associations between neighborhood characteristics and dating violence: does spatial scale matter?

**DOI:** 10.1186/s12942-022-00306-3

**Published:** 2022-06-20

**Authors:** Paul Rodrigues, Martine Hébert, Mathieu Philibert

**Affiliations:** Département de Sexologie, Université du Québec à Montréal, Succursale Centre-Ville, Case postale 8888, Montréal, Québec H3C 3P8 Canada

## Abstract

**Background:**

Dating violence (DV) is a public health problem that could have serious repercussions for the health and well-being of a large number of adolescents. Several neighborhood characteristics could influence these behaviors, but knowledge on such influences is still limited. This study aims at (1) evaluating the associations between neighborhood characteristics and DV, and (2) assessing how spatial scale influences the estimations of the latter associations.

**Methods:**

The Québec Health Survey of High School Students (2016–2017) was used to describe DV. Neighborhoods were operationalized with polygon-based network buffers of varying sizes (ranging from 250 to 1000 m). Multiple data sources were used to describe neighborhood characteristics: crime rate, alcohol outlet density (on-premises and off-premises), walkability, greenness, green spaces density, and youth organizations density. Gendered-stratified logistic regressions were used for assessing the association between neighborhood characteristics and DV.

**Results:**

For boys, off-premises alcohol outlet density (500 m) is associated with an increase in perpetrating psychological DV. Crime rate (500 m) is positively associated with physical or sexual DV perpetration, and crime rate (250 m) is positively associated with physical or sexual DV victimization. Greenness (1000 m) has a protective effect on psychological DV victimization. For girls, walkability (500 m to 1000 m) is associated with a decrease in perpetrating and experiencing psychological DV, and walkability (250 m) is negatively associated with physical or sexual DV victimization.

**Conclusions:**

Several neighborhood characteristics are likely to influence DV, and their effects depend on the form of DV, gender, and spatial scale. Public policies should develop neighborhood-level interventions by improving neighborhood living conditions.

**Supplementary Information:**

The online version contains supplementary material available at 10.1186/s12942-022-00306-3.

## Introduction

Teen dating violence (DV), which can be described as psychologically, physically, or sexually abusive behaviors from a dating partner, is a major public health problem. A recent meta-analysis estimated an overall prevalence of 20% (ranging from 1 to 61%) for physical victimization and 9% (ranging from < 1 to 54%) for sexual victimization [1]. Psychological violence was not assessed in this meta-analysis, but it appears to be the most common form of DV. A systematic review suggests that the prevalence of psychological violence ranges from 17 to 88% [2]. In addition to its high prevalence, DV is associated with negative repercussions on the health and well-being of victims, such as anxiety, depression, post-traumatic stress symptoms, as well as suicidal ideation [3–5].

Research on determinants of DV has mainly focused on individual (e.g., antisocial or risky behaviors), family (e.g., exposure to family violence, lack of parental supervision), and peer (e.g., affiliation with deviant peer) factors [6–8]. Empirical analyses of the association between neighborhood characteristics and DV are scarce. Among studies exploring such an association, most studies analyzed the effect of neighborhoods’ characteristics such as the sociodemographic composition, neighborhood disorder (i.e., visible social (e.g., crime) and physical (e.g., vandalism) signs of decay), collective efficacy and access to alcohol outlets, and led to inconsistent findings [9]. Yet, characteristics of the physical environment are seldom analyzed in relation to DV despite reports of associations with adolescents’ behaviors, possibly reflecting socioenvironmental and psychobehavioral mechanisms. For example, neighborhood greenness, walkability, access to green spaces, and access to community organizations could enhance social cohesion and reduce adolescents’ aggression [10–12], which could positively affect DV [7–9]. However, to our knowledge, the influence of these physical environment factors on DV has never been assessed.

Furthermore, previous studies on the neighborhood effects on DV have used census tracts to operationalize neighborhoods. There is little discussion of how to operationalize neighborhoods and the scale of analysis, but these choices could potentially influence the estimation of the effect of neighborhood factors [13]. In addition, administrative units, such as census tracts, may not accurately reflect individual experience of space. Egocentric neighborhoods, defined as a buffer around a location, such as individuals’ homes, could better address these limitations [14–16], but this approach is little used in research on DV. Against this backdrop, the current study aimed to analyze a range of neighborhood characteristics possibly related to DV, many of which have not yet been explored, and explore the effects of the spatial scale of analysis.

## Context

### Neighborhood risk factors and DV

The relationship between neighborhood characteristics and DV has been explored through social disorganization theory focusing on neighborhood-level risk factors [9]. This theory posits that violence and criminality are more likely to occur in socially disorganized neighborhoods due to the community’s inability to collectively manage problems within the neighborhood [17]. Such context could also foster neighborhood disorder [17, 18] and exacerbate negative consequences of alcohol outlets (e.g., criminality, alcohol abuse) [19, 20], thereby influencing individuals’ violent behaviors.

Several empirical analyses assessed the effects of neighborhood disorder and density of alcohol outlets on DV, but results are mixed. Some studies found positive associations between perceived neighborhood disorder and DV [21–24], while others reported no significant associations between crime rate, a dimension of neighborhood disorder, and DV [25, 26]. The association between alcohol outlet density and DV has been observed among young adults [27–29], but studies have yet to assess this relationship among adolescents. However, alcohol outlet density could have a different effect on this population as selling alcohol to minors is prohibited.

Different mechanisms could explain the effect of neighborhood disorder and access to alcohol outlets on DV. Neighborhood disorder is often incorporated into social disorganization theory, which describes it as a marker of the lack of order in the community and the inefficiency of social control [30, 31]. Disorder may refer to minor offences (e.g. graffiti, vandalism) which may lead to more serious crimes and create fear and distrust among residents [31]. Such conditions could encourage violent behaviors, including DV [9]. Exposure to neighborhood violence, a component of disorder sometimes used to measure the effect of neighborhood disorder, may also come to foster the normalization of violence and increase frustration and anger in adolescents, which may influence the adoption of such behaviors [9].

The density of alcohol outlets in neighborhoods is a potential risk factor for many violent behaviors, including interpersonal violence. The presence of alcohol outlets provides opportunities for consumption [32], which could result in risky drinking behaviors [19]. Alcohol abuse could, in turn, increase the risk of interpersonal violence victimization [8] and perpetration [7]. In socially disorganized neighborhoods, alcohol outlet density may also enhance neighborhood disorder by encouraging the gathering of people likely to adhere to norms and attitudes conducive to alcohol consumption and violence [19].

### Neighborhood protective factors and DV

While neighborhood disorder and alcohol outlet density could negatively impact adolescents’ behaviors, some environmental and institutional resources could be associated with positive effects [33]. In particular, the level of greenness, the density of green spaces, the density of community organizations and walkability could prevent DV through several mechanisms.

Greenness and accessibility to green spaces could affect some physiological and psychosocial processes, which in turn could influence DV. According to the biophilia hypothesis, exposure to nature may improve mental health by restoring cognitive functions and reducing mental fatigue [34]. A higher level of greenness in neighborhoods may also be associated with a lower risk of depressive symptoms [35, 36] and aggressivity [37] in adolescents, both of which have been identified as potential risk factors of DV perpetration [7] as well as victimization [8]. A study on intimate partner violence also found that rates of aggression were lower in neighborhoods characterized by a high level of greenness [38]. Access to green spaces, such as public parks, could promote participation in physical and recreational activities and encourage social interactions [39]. Physical activity may improve self-esteem and have a protective effect on the onset of depression and anxiety [40], all three determinants of DV [7, 8]. Social interactions may increase social cohesion in neighborhoods [41], which is likely to reduce the risk of DV [42, 43].

Geographic access to community organizations (e.g., social clubs, sports clubs) may promote adolescents’ participation in structured and supervised activities [10, 44]. Such resources provide opportunities to develop social ties between adolescents as well as between residents of the neighborhood (e.g., their parents) [10]. Community organizations are also safe places that could reduce exposure to the negative aspects of neighborhoods, such as disorder [10, 45] and foster participation in supervised activities. Thus, adolescents attending these facilities could have a lower risk of risky behaviors, such as substance use [10, 46] and delinquency [46–48], which are determinants of DV [7, 8].

Walkability refers to the attributes of urban design (e.g., road network, land use) promoting walking [49]. Walkability is likely to promote social interactions, which could contribute to a better sense of belonging [11, 50] and collective efficacy (i.e., the ability of the community to act collectively to regulate deviant behaviors) [11]. These concepts are central to social disorganization theory and could influence DV [9]. To our knowledge, the effect of walkability on DV has not been described. However, empirical studies suggest that increased walkability is associated with a decrease in homicide [51] and violent behaviors [52] among adolescents.

Finally, it is essential to note that the associations between neighborhood characteristics and DV may be modified by gender. Most studies suggest that neighborhood effects are stronger for boys than for girls [53, 54]. Boys may have less parental supervision than girls [55], leading to greater exposure to their neighborhood [56, 57].

### Defining neighborhoods

In addition to the lack of knowledge about neighborhood effects on DV, previous studies have not explored the impact of neighborhoods’ operationalization and spatial scale of the estimated effects. Most studies on DV used census tracts to measure the level of crime [25, 26] and the density of alcohol outlets [27–29], but this choice remains little discussed, despite being known to geographers to affect statistical analyses.

Two biases are related to neighborhoods’ operationalization: the Modifiable Areal Unit Problem (MAUP) and the Uncertain Geographic Context Problem (UGCoP). The MAUP suggests that the shape and size of spatial units could lead to variations in the estimated effects of neighborhood characteristics [58, 59]. The UGCoP argues that the estimation of neighborhood effects may be affected by the definition of neighborhoods and the use of inappropriate spatial units [60]. Both biases could limit the ability to observe associations between neighborhood characteristics and a given outcome. For example, census tracts were used in previous studies on DV even though they may not be an adequate representation of neighborhood-level influences. The MAUP and UGCoP could also partly explain why the effect of certain factors, such as crime [25, 26], has not yet been observed.

In effect, administrative units, such as census tracts, may not adequately represent individuals' actual exposure to their neighborhood. These units have artificial boundaries, which implicitly assume that individuals’ environments are limited to the corresponding spatial units, regardless of their real location inside the spatial unit. However, individuals living closely to each other but in two different spatial units could have more similar exposures to neighborhood factors than two individuals living further away but in the same spatial unit. Neighborhood-level processes, such as social interactions or access to resources, may depend on geographical proximity and can be observed across these boundaries [14, 16, 61]. In consequence, the accuracy of measures of exposure to neighborhood factors could vary depending on the location of individuals within a spatial unit. These measures could be less accurate for people living further away from the centroid of the spatial unit [62–64]. When geographic access to resources is assessed, using large spatial units such as census tracts could also lead to aggregation error [62, 63]. In response to such a caveat, egocentric neighborhoods were proposed as operational forms of neighborhoods which allow for integrating proximity. They provide a person-centered approach and refer to the set of places located within a given distance from the individuals’ homes [15, 16, 65]. Egocentric neighborhoods are defined specifically for each individual and allow for overlapping neighborhoods, suggesting that some individuals share common exposures to neighborhood factors depending on their proximity. An egocentric definition of neighborhoods could more adequately reflect the real use of spaces by residents and their perception of the residential context [14, 16, 61].

## Objectives

The current study had two main objectives: (1) to analyze the association between several contextual characteristics (crime, density of off-premises alcohol outlets, density of bars, density of community organizations, level of greenness, density of green spaces and walkability) and DV (victimization and perpetration) and (2) to assess scale effects in studying these relationships.

## Data and method

### Participants

**Fig. 1:**
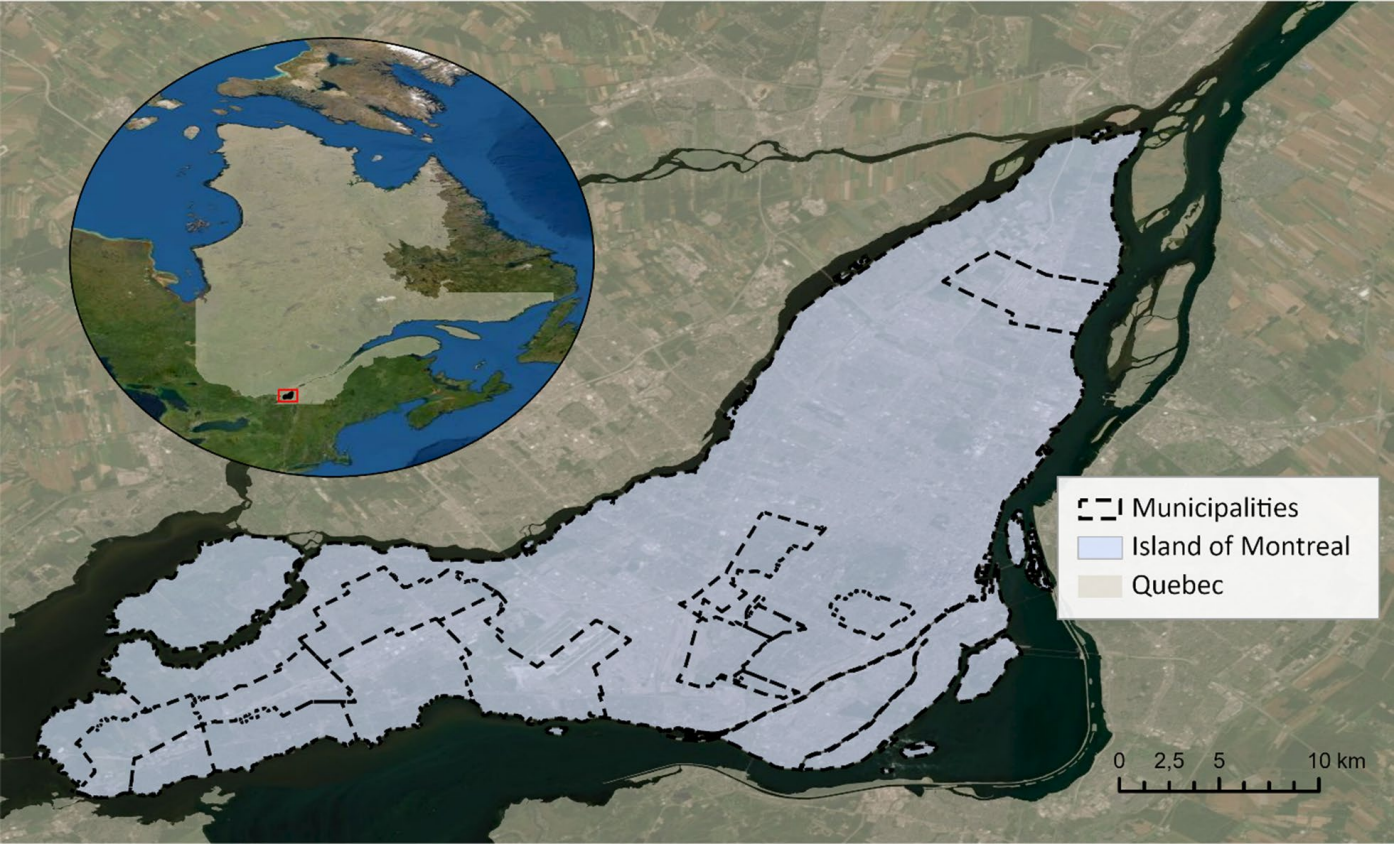
Study area-island of Montréal

The current study used data from the Québec Health Survey of High School Students (QHSHSS) 2016–17, a cross-sectional survey of secondary-school Québec youth (Grades 7 to 11). A three-stage stratified cluster sampling was used to recruit participants in this survey. Schools were randomly selected for each grade level and each health region separately. Classes were randomly selected from the selected schools. The QHSHSS provides a representative sample for the province of Québec and each health region. Only participants whose postal code of residence was located in the island of Montréal were included in this study (*n* = 2,687). The island of Montréal had a population of 1,942,040 inhabitants in 2016 (about 23.8% of the population of the province of Québec, Canada) and is composed of 15 municipalities (Fig. 1) [66]. About 85% of the island's population lives in the city of Montréal, the largest city in Québec and the second largest in Canada.

### Dependent variables

Four variables describing different forms of DV were used. Two variables referred to victimization (e.g., “He/she (…) me”), and two referred to perpetration (e.g., “I (…) him/her).

Psychological DV was assessed by items derived from two questions. One item referred to verbal violence (“I criticized him/her viciously about his/her physical appearance; I insulted him/her in front of people; I put him/her down.” / “He/she viciously criticized my physical appearance; he/she insulted me in front of people; he/she put me down.”), while the other related to controlling behaviors (“I controlled his/her outings, email conversations or cell phone; I prevented him/her from seeing his/her friends.” / “He/she controlled my outings, my email conversations or cell phone; he/she prevented me from seeing my friends”). The response scale ranged from 0 (never during the past 12 months) to 3 (three times or more during the past 12 months). A dichotomous measure was obtained by distinguishing participants who had experienced one of these events at least once (one or more) from those who reported having never experienced these situations.

To assess physical violence, four items from the *Conflict Tactics Scale* [67] (e.g., “I slapped him/her”/ “He/She slapped me”) were used. Sexual violence was assessed by two items referring to experiences of sexual activity without consent (e.g., “I forced him/her to have sexual contact or sexual intercourse with me when he/she didn’t want to” / “He/she forced me to have sexual contact or sexual intercourse when I didn’t want to.”). Due to the small number of participants who reported sexual violence perpetration, physical and sexual DV were merged. Dichotomous measures were derived by differentiating participants who had experienced physical or sexual violence at least once from those who had never experienced these situations.

### Neighborhood-level variables

#### Egocentric neighborhoods

**Fig. 2:**
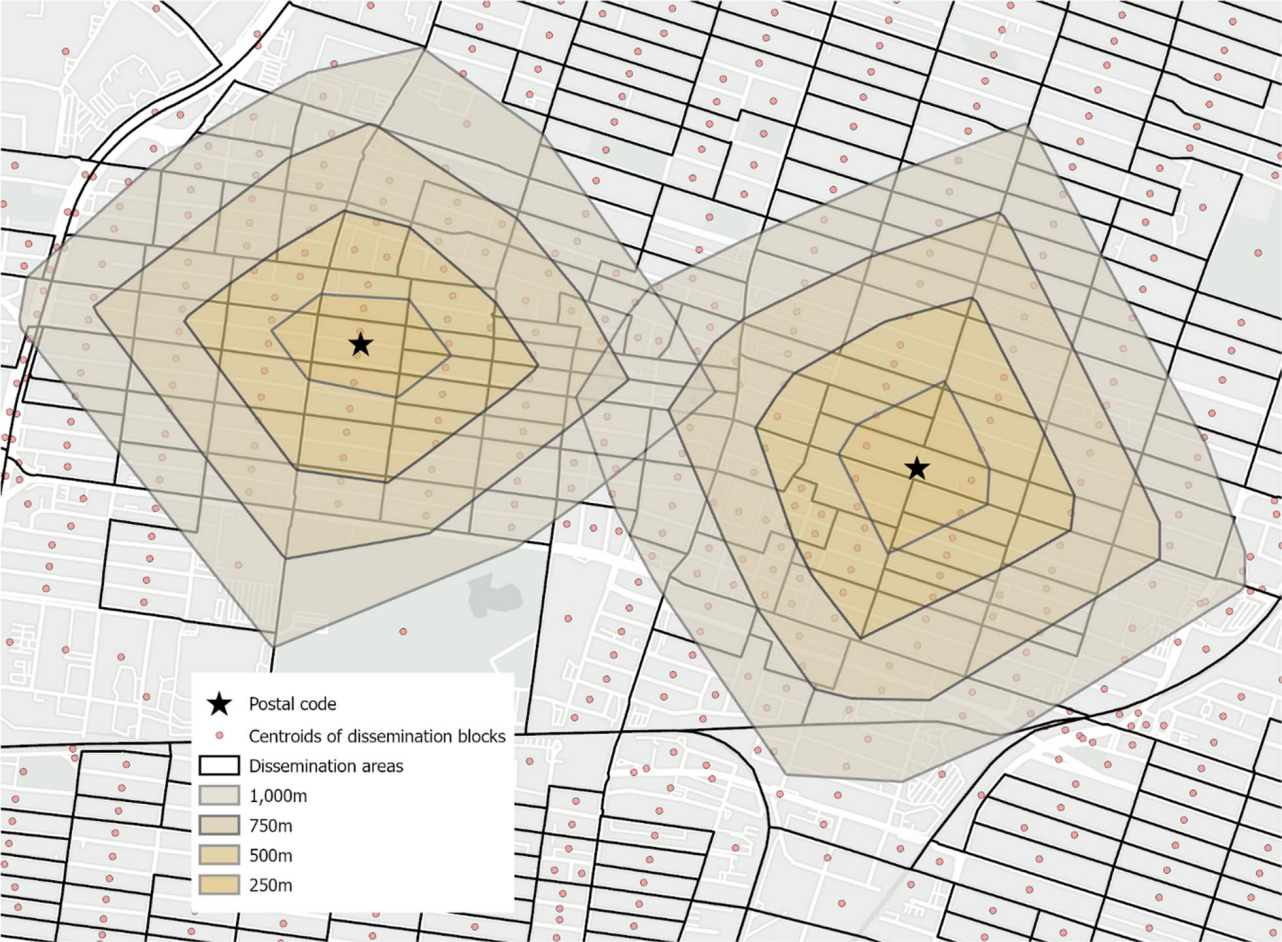
Egocentric neighborhoods

Egocentric neighborhood refers to the area within a specific radius around the individual’s home and is often operationalized using buffer zones [15, 16, 65]. In this study, the participants' place of residence was estimated from the centroid of the postal code area. Postal codes are managed by Canada Post for mail delivery and have an average of 14.5 dwellings in Montréal. Their centroids provide a good approximation of the exact address of participants [68]. Egocentric neighborhoods were operationalized from polygon-based network buffers around the participants’ place of residence, a method of buffering providing an accurate representation of the spatial area used by individuals [65]. In this study, the government of Québec’s official road network database (Addresses Québec) and the Network Analyst extension of ArcGIS Pro 2.7.0 were used to generate all routes from participants’ place of residence to a specific distance. Polygon-based network buffers were then computed by connecting the endpoints of these routes, resulting in irregular polygons (Fig. 2). For each participant, four buffer zones were created using different distances: 250 m, 500 m, 750 m, and 1000 m. Figure 2 provides an example of polygon-based network buffers for two postal codes.

#### Criminality

The level of crime was measured using data from Service de police de la Ville de Montréal (SPVM) for the years 2016 and 2017. This database provides the date and the location of crime events at the nearest intersection. Events pertaining to one of the following six categories were retained: offences resulting in death, intrusion, mischief, theft in/on a vehicle, motor vehicle theft, and robbery. First, the geolocation of crime events allowed their number to be calculated for each egocentric neighborhood (buffer zone). Secondly, the population size of each egocentric neighborhood was estimated by summing the population of dissemination blocks (DB) included in the neighborhood. DBs are the smallest spatial units for which data on population size are disseminated by Statistics Canada. If only part of the DBs was included in a buffer, only the proportion of its population corresponding to the proportion of area included in the neighborhood was considered for summing the population. The crime rate is the number of crimes divided by twice the population size (the number of crimes was estimated for two years) multiplied by 100.

#### Greenness and access to green space

The Normalized Difference Vegetation Index (NDVI) is commonly used in epidemiological studies and provides an objective and accurate measure of overall greenness [69]. This index was first produced at a 30 × 30 m spatial resolution from Landsat 8 satellite data (United States Geological Survey, 2015–2016). The greenness of the egocentric neighborhoods was estimated using the average NDVI in the area covered by a buffer.

To assess access to green spaces, a map of public parks and green spaces was first obtained for the island of Montréal by cross-referencing information provided by the municipalities. The municipalities of the City of Montréal, Dollard-des-Ormeaux, Pointe-Claire, Kirkland, and East Montréal provided the location of parks and green spaces as open access data. A map of parks and green spaces was created for the remaining cities using land use data at the parcel-level from the property assessment roll provided by the Montréal Metropolitan Community (2016) and information available on the municipalities' websites. The parcels on the property assessment roll were manually identified as green space based on the documents and maps available on the websites. A total of 1389 green spaces were identified for the island of Montréal. Access to green spaces was estimated by the number of green spaces that intersected each buffer.

#### Access to alcohol outlets

Alcohol outlets were located using data on private outlets licensed by the *Régie des alcools, des courses et des jeux* (RACJ, 2016) and outlets administered by the *Société des alcools du Québec* (SAQ, 2016). A total of 1432 bars and 1842 off-premises alcohol outlets (e.g., convenience stores, grocery shops and SAQ outlets) were identified from these data. Using the same method as for green spaces, density measures have been used to estimate access to bars and access to off-premises alcohol outlets separately.

#### Access to community organizations

The Directory of Community Organisations from 211, an information and referral public service, was used to identify and locate community organizations for young people (e.g., youth centers, YMCA, sports associations). Lists of community organizations in the different municipalities of the island of Montréal, which are available on the corresponding websites, supplemented these data. A total of 423 community organizations offering services for young people were identified. To assess access to community organizations, density measures were used by calculating the number of community organizations within each buffer.

#### Walkability

For each egocentric neighborhood, walkability was measured using three variables: land use mix, residential density, and intersection density. Firstly, data from the property assessment roll (parcel-level land use) provided by the Montréal Metropolitan Community (2016) was used to assess the land use mix. An entropy index [70] was elaborated to describe the level of heterogeneity in land use considering four categories of land use: residential, commercial, services and cultural, recreational and leisure. Secondly, data from the property assessment roll were used to calculate the net residential density. This variable refers to the number of dwellings per hectare of the residential area included in each buffer. Thirdly, data from Addresses Québec (2016) were used to calculate the density of intersections with three or more segments of the road network (excluding highways). The walkability measure used in this study is based on several studies reporting on the development of this index [71, 72]. It was obtained by the sum of the z-scores of all variables described above: z-*score(land use mix)* + *z-score(residential density)* + *1.5(z-score(intersection density))*. In its complete form, the walkability index also requires the retail floor area ratio [71], which could not be obtained as is the case for many studies. The weight of 1.5 for intersection density (replacing a weight of 2 in the complete index) was proposed by Sundquist et al. [72] as a way to compensate for the lack of data on retail floor area. This walkability index has been validated and is a good predictor of walking behaviors [72]. A study also found a strong correlation between the three-components index and the four-components index, and both were predictors of utilitarian walking [73], confirming the validity of using the three-components walkability index as a surrogate for the four-components index.

### Covariates

#### Individual-level covariates

Several individual-level covariates were used: gender (girl or boy), high school grade level (Grades 7 or 8, 9, 10, and 11), the highest level of parental education (high school or less, college or professional training, university), family structure (two parents, blended family, or shared custody, living with one parent or other family structure), and parental country of birth (two parents born in Canada, at least one parent born outside Canada).

#### Neighborhood sociodemographic characteristics covariates

Five potentially confounding neighborhood sociodemographic characteristics were measured using data from the 2016 Canadian census.

Population density corresponded to the number of inhabitants per hectare and was estimated from the population size and the area of the buffers. The population size of each egocentric neighborhood was obtained by summing the population size of the DBs (i.e., the smallest spatial units for which population size is available) within the corresponding buffer zone weighted by the proportion of the area of the included DBs (a weight of 1 was used for DBs completely located in the buffer).

Although population size data are available at the DB-level, no sociodemographic data are disseminated by Statistics Canada at this scale. For this reason, the four remaining sociodemographic variables were derived from variables at the dissemination area (DA) level, the smallest spatial units for which sociodemographic data are available. As DBs are embedded into DAs (see Fig. 2), the sociodemographic measures were assigned to the corresponding DBs, assuming a homogeneous distribution within each DA. Similar to the method used to estimate population density, each egocentric neighborhood was composed of the DBs included in the corresponding buffer, weighted by the proportion of the area of included DBs.

Socioeconomic status (SES) was measured by median income. For each egocentric neighborhood, the median income was estimated by calculating the population-weighted median of the DBs included in the corresponding buffer. Median income was used as it is less sensitive to extreme values than average income and may therefore better capture the spatial distribution of income.

The percentage of single-parent dwellings was used to assess single parenthood. The number of dwellings and the number of single-parent dwellings were estimated for each egocentric neighborhood by summing the frequencies of the two variables at the DB-level.

Residential instability was measured using the percentage of residents living in the egocentric neighborhood for five years or less. For each egocentric neighborhood, the number of residents living there for less than five years and the population size were estimated by summing the frequencies of the DBs considered as part of the corresponding buffer.

Finally, to assess ethnocultural diversity, a language diversity index was developed. Languages spoken at home were divided into 16 classes based on the World Value Survey classification [74]. Frequencies of each class were first obtained for each egocentric neighborhood by summing the frequencies of the corresponding DBs. Shannon entropy index [70] was then calculated for each egocentric neighborhood to estimate the level of heterogeneity/homogeneity of languages spoken at home.

### Statistical analysis

**Fig. 3:**
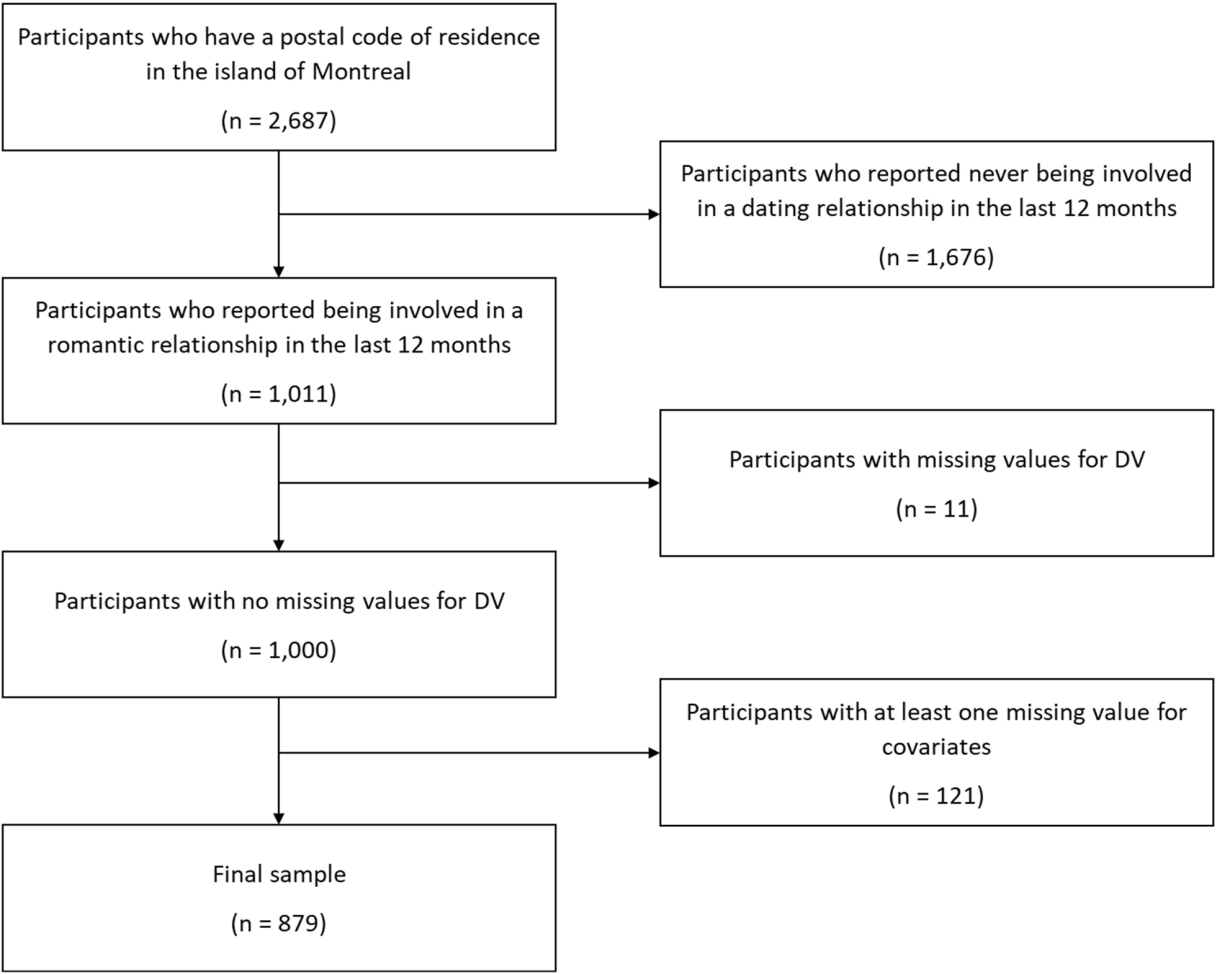
Flowchart for sample selection

Data at the neighborhood level were matched with data from the QHSHSS at the individual level using participants’ postal codes. Only adolescents who reported being involved in a romantic relationship in the last 12 months and had no missing values for the DV measures were included (37% of participants with a postal code in Montréal). Among them, 121 had at least one missing value for covariates and were excluded (12% of participants who reported a romantic relationship). The final sample consisted of 879 adolescents (Fig. 3).

The associations between neighborhood characteristics and DV were estimated with logistic regressions using the SURVEYLOGISTIC procedure of SAS Enterprise Guide 8.5 [75]. All models considered the sampling design and used bootstrap weights. They were carried out separately for girls and boys to account for gender differences. A Directed Acyclic Graph (DAG) was developed to identify confounding variables (Additional file 1). A DAG is a graphical representation of causal assumptions regarding a set of variables and can be used as a tool to identify confounders [76, 77]. This approach can also limit the risk of overadjustment bias [77]. This bias occurs when a model controls for variables that are not confounders. Assessing neighborhood effects could be subject to overadjustment bias due to the complex relationships and high correlations between neighborhood factors. The DAG used in the current study thus provided a parsimonious approach and allowed the identification of confounding factors tailored to each neighborhood factor specifically. Based on this DAG, not all models required the same set of covariates. Associations between neighborhood characteristics and DV variables were estimated using different models in three stages. (1) Associations between DV and walkability were estimated using models including individual-level variables and neighborhood sociodemographic characteristics as covariates. (2) Associations between DV and the level of greenness, the density of green spaces, the density of community organizations, the density of off-premises alcohol outlets, and the density of bars were estimated by controlling for individual-level variables, neighborhood sociodemographic characteristics as well as walkability. (3) Associations between DV and neighborhoods’ level of crime were modelled controlling for all the variables described above, except for the density of community organizations.

At each stage and for each dependent variable, four models estimated the association with a given neighborhood characteristic at different scales, i.e. using different buffer sizes (250 m, 500 m, 750 m, and 1000 m). For each of these models, covariates were modelled at the scale considered the most appropriate in the previous stage. Choosing the most appropriate scale for modelling a given neighborhood characteristics was based on the comparison of the models' fit using the Akaike Information Criterion (AIC). The selected scale was the one with the lowest AIC. It should be noted that the scale for neighborhood sociodemographic characteristics used as covariates in all models was similarly identified in preliminary analyses. In these analyses, the effect of neighborhoods’ sociodemographic characteristics on the DV measures were estimated separately in models adjusted by individual-level variables only (results available upon request). For example, for perpetration of psychological DV among boys, preliminary analyses showed that the most appropriate scale for neighborhood sociodemographic characteristics (lowest AIC) were 1000 m for median income, 250 m for percentage of single-parent dwellings, 500 m for residential instability, 250 m for ethnocultural diversity, and 1000 m for population density. These variables were used in Stage 1 to estimate the effect of walkability. In this stage, models showed that walkability within 250 m had the best fit (lowest AIC). Variables selected in preliminary analyses and Stage 1 were then used to estimate the effects of all variables identified in Stage 2. In Stage 3, to assess the effect of crime rate, the scales used for neighborhood characteristics covariates were based on results in all previous stages. This method provided parsimonious estimations of neighborhood factors for each outcome. Multicollinearity was assessed for all models using Variance Inflation Factors (VIF) and the data showed no multicollinearity problem (VIF < 4).

In line with the second objective of this study, the influence of the spatial scale of analysis was evaluated by comparing the AIC of models estimating associations between the same neighborhood-level factors and a given outcome across buffers (250 m, 500 m, 750 m, and 1000 m). As a rule of thumb, an AIC difference greater than two units suggest that the model with the lowest AIC is the most predictive [78]. Scale effects were also investigated by calculating Spearman’s rho correlation coefficients between all neighborhood-level variables across buffers. Weak correlations suggested differences in measurements of neighborhood-level variables across buffers, while strong correlations suggested minor differences. Spearman’s correlations were used instead of Pearson’s correlations because some variables were skewed.

## Results

### Sample description

Most participants reported living in a two-parent family (64.74%) with at least one parent who has obtained a university degree (68.56%) (Table 1). Psychological violence was the most prevalent form of DV: 20.25% of adolescents reported perpetration, while 28.78% reported experiences of victimization. Perpetration and victimization of physical/sexual DV were observed respectively by 14.10% and 19.94% of participants. For all forms, girls were more likely to report an experience of DV.

**Table 1:** Descriptive statistics

	All (n = 879)	Girls (n = 452)	Boys (n = 427)
Individual-level variables	%	%	%
Psychological DV perpetration			
At least once	20.25	23.76	16.68
Never	79.75	76.24	83.32
Physical or sexual DV perpetration			
At least once	14.10	20.41	7.67
Never	85.90	79.59	92.33
Psychological DV victimization			
At least once	28.78	34.13	23.33
Never	71.22	65.87	76.67
Physical or sexual DV victimization			
At least once	19.94	22.83	17.01
Never	80.06	77.17	82.99
Grade level			
Grade 7 or 8	29.09	25.09	33.15
Grade 9	18.32	18.06	18.58
Grade 10	23.71	23.93	23.50
Grade 11	28.88	32.92	24.77
Parental country of birth			
Two parents born in Canada	42.21	37.88	46.61
At least one parent born outside Canada	57.79	62.12	53.39
Family structure			
Two parents	64.74	61.96	67.57
Blended family or shared custody	16.91	19.13	14.66
Living with one parent or other family structure	18.35	18.92	17.78
Highest level of parental education			
High school or less	13.06	16.55	9.50
College or professional training	18.38	20.20	16.54
University	68.56	63.25	73.96

Neighborhood-level variables	Mean (SD)	Mean (SD)	Mean (SD)
*250 m*			
Sociodemographic characteristics			
Median income	58,754.92 (25,196.54)	57,754.87 (22,883.30)	59,772.37 (27,339.35)
Single parenthood	20.32 (7.63)	20.72 (7.40)	19.92 (7.84)
Residential instability	38.94 (13.13)	39.39 (12.84)	38.48 (13.42)
Ethnocultural diversity	1.28 (0.39)	1.30 (0.39)	1.27 (0.39)
Population density	9.89 (6.07)	9.90 (5.88)	9.88 (6.25)
Risk factors			
Density of off-premises alcohol outlets	0.75 (1.18)	0.74 (1.12)	0.76 (1.23)
Density of bars	0.31 (1.00)	0.35 (1.03)	0.28 (0.97)
Crime rate	1.54 (2.18)	1.64 (2.76)	1.44 (1.34)
Protective factors			
Walkability	**−** 0.47 (2.61)	**−** 0.37 (2.57)	**−** 0.58 (2.65)
NDVI	0.50 (0.11)	0.49 (0.11)	0.50 (0.11)
Density of green spaces	0.87 (0.95)	0.89 (0.92)	0.86 (0.99)
Density of community organizations	0.39 (0.65)	0.37 (0.64)	0.40 (0.67)
*500 m*			
Sociodemographic characteristics			
Median income	57,714.61 (22,067.14)	56,744.45 (20,220.35)	58,701.65 (23,782.52)
Single parenthood	20.33 (6.56)	20.74 (6.43)	19.91 (6.67)
Residential instability	39.37 (11.88)	39.67 (11.50)	39.06 (12.26)
Ethnocultural diversity	1.29 (0.37)	1.30 (0.38)	1.27 (0.37)
Population density	8.61 (4.42)	8.65 (4.41)	8.58 (4.44)
Risk factors			
Density of off-premises alcohol outlets	3.26 (3.91)	3.24 (3.77)	3.27 (4.04)
Density of bars	1.50 (3.63)	1.51 (3.04)	1.49 (4.14)
Crime rate	1.32 (3.16)	1.24 (1.01)	1.41 (4.37)
Protective factors			
Walkability	**−** 0.53 (2.66)	**−** 0.44 (2.62)	**−** 0.61 (2.69)
NDVI	0.50 (0.10)	0.49 (0.10)	0.50 (0.10)
Density of green spaces	2.86 (2.35)	2.9 (2.32)	2.82 (2.38)
Density of community organizations	1.10 (1.24)	1.06 (1.15)	1.15 (1.31)
*750 m*			
Sociodemographic characteristics			
Median income	56,871.79 (19,941.78)	56,039.25 (18,387.81)	57,718.81 (21,395.60)
Single parenthood	20.52 (5.86)	20.87 (5.73)	20.16 (5.97)
Residential instability	39.68 (11.01)	39.97 (10.5)	39.40 (11.52)
Ethnocultural diversity	1.29 (0.36)	1.31 (0.36)	1.28 (0.36)
Population density	7.99 (3.80)	8.09 (3.79)	7.89 (3.81)
Risk factors			
Density of off-premises alcohol outlets	7.32 (7.64)	7.29 (7.56)	7.36 (7.72)
Density of bars	3.78 (7.98)	3.83 (7.55)	3.72 (8.41)
Crime rate	1.18 (0.64)	1.18 (0.63)	1.19 (0.65)
Protective factors			
Walkability	**−** 0.55 (2.68)	**−** 0.46 (2.63)	**−** 0.65 (2.74)
NDVI	0.49 (0.10)	0.49 (0.10)	0.50 (0.10)
Density of green spaces	5.75 (4.18)	5.84 (4.29)	5.66 (4.07)
Density of community organizations	2.08 (1.89)	2.00 (1.81)	2.16 (1.97)
*1000 m*			
Sociodemographic characteristics			
Median income	56,237.1 (17,931.19)	55,576.33 (16,738.62)	56,909.36 (19,064.11)
Single parenthood	20.65 (5.40)	20.95 (5.34)	20.34 (5.45)
Residential instability	40.08 (10.44)	40.39 (9.93)	39.76 (10.94)
Ethnocultural diversity	1.30 (0.35)	1.31 (0.35)	1.29 (0.34)
Population density	7.51 (3.40)	7.60 (3.35)	7.42 (3.45)
Risk factors			
Density of off-premises alcohol outlets	12.69 (12.60)	12.73 (12.72)	12.65 (12.49)
Density of bars	6.95 (13.98)	7.05 (13.57)	6.85 (14.40)
Crime rate	1.18 (0.58)	1.18 (0.58)	1.18 (0.59)
Protective factors			
Walkability	**−** 0.54 (2.69)	**−** 0.44 (2.63)	**−** 0.64 (2.75)
NDVI	0.49 (0.09)	0.49 (0.09)	0.49 (0.09)
Density of green spaces	9.42 (6.38)	9.53 (6.60)	9.31 (6.15)
Density of community organizations	3.42 (2.75)	3.29 (2.72)	3.54 (2.78)

### Models of associations between neighborhood characteristics on DV among boys

Table 2 summarizes the results from logistic regressions analyzing the relationship between neighborhood characteristics and DV among boys.

**Table 2:** Associations between neighborhood characteristics and DV among boys

	250 m		500 m		750 m		1000 m	
	ß (SE)	AIC	ß (SE)	AIC	ß (SE)	AIC	ß (SE)	AIC
**Psychological DV perpetration**								
Risk factors								
Density of off-premises alcohol outlets^2^	0.15 (0.14)	363.351	0.08 (0.04)*	362.064	0.05 (0.03)	362.621	0.02 (0.02)	363.195
Density of bars^2^	**−** 0.18 (0.17)	363.249	**−** 0.03 (0.03)	363.620	**−** 0.02 (0.01)	363.449	**−** 0.01 (0.01)	363.380
Crime rate^3^	**−** 0.08 (0.13)	362.791	**−** 0.21 (0.34)	362.638	**−** 0.56 (0.35)	361.415	**−** 0.33 (0.39)	362.628
Protective factors								
Walkability^1^	0.09 (0.08)	362.323	0.10 (0.09)	362.486	0.09 (0.09)	362.629	0.10 (0.09)	362.365
NDVI^2^	**−** 1.92 (2.29)	363.504	**−** 1.00 (2.37)	364.134	**−** 0.89 (2.53)	364.187	**−** 0.45 (2.54)	364.289
Density of green spaces^2^	**−** 0.28 (0.21)	361.705	**−** 0.07 (0.08)	363.399	**−** 0.06 (0.05)	362.343	**−** 0.04 (0.03)	362.239
Density of community organizations^2^	0.24 (0.23)	362.165	**−** 0.02 (0.12)	364.297	**−** 0.07 (0.08)	363.611	**−** 0.03 (0.06)	364.085
**Physical or sexual DV perpetration**								
Risk factors								
Density of off-premises alcohol outlets^2^	0.01 (0.17)	242.997	0.04 (0.06)	242.771	0.00 (0.03)	243.000	0.01 (0.03)	242.950
Density of bars^2^	**−** 0.32 (0.26)	241.920	**−** 0.03 (0.06)	242.897	**−** 0.03 (0.02)†	242.115	**−** 0.02 (0.01)	242.270
Crime rate^3^	0.14 (0.16)	245.851	0.97 (0.28)***	237.656	0.84 (0.37)*	241.587	0.08 (0.56)	246.688
Protective factors								
Walkability^1^	0.04 (0.12)	241.001	**−** 0.02 (0.11)	241.101	0.02 (0.11)	241.090	**−** 0.01 (0.12)	241.117
NDVI^2^	0.86 (3.30)	242.908	2.54 (3.95)	242.334	2.43 (4.11)	242.470	1.77 (3.86)	242.728
Density of green spaces^2^	0.10 (0.17)	242.730	**−** 0.04 (0.06)	242.811	**−** 0.03 (0.05)	242.744	**−** 0.03 (0.04)	242.436
Density of community organizations^2^	0.04 (0.36)	242.984	**−** 0.15 (0.18)	242.313	**−** 0.11 (0.16)	242.264	**−** 0.05 (0.11)	242.714
**Psychological DV victimization**								
Risk factors								
Density of off-premises alcohol outlets^2^	0.17 (0.13)	460.258	0.06 (0.05)	460.447	0.01 (0.03)	461.867	0.00 (0.02)	462.022
Density of bars^2^	0.01 (0.11)	462.040	**−** 0.03 (0.03)	461.575	**−** 0.01 (0.01)	461.414	**−** 0.01 (0.01)	461.505
Crime rate^3^	0.00 (0.11)	461.795	**−** 0.01 (0.03)	461.770	**−** 0.13 (0.35)	461.644	**−** 0.17 (0.42)	461.612
Protective factors								
Walkability^1^	**−** 0.05 (0.08)	461.103	**−** 0.10 (0.09)	460.043	**−** 0.08 (0.10)	460.752	**−** 0.01 (0.09)	461.562
NDVI^2^	**−** 2.75 (1.99)	459.792	**−** 2.88 (2.13)	460.042	**−** 3.59 (2.13)†	459.257	**−** 4.24 (2.09)*	458.285
Density of green spaces^2^	**−** 0.22 (0.16)	459.432	**−** 0.01 (0.05)	462.005	0.00 (0.03)	462.042	**−** 0.01 (0.03)	461.718
Density of community organizations^2^	0.25 (0.22)	460.124	0.01 (0.11)	462.023	**−** 0.01 (0.08)	462.007	**−** 0.02 (0.06)	461.913
**Physical or sexual DV victimization**								
Risk factors								
Density of off-premises alcohol outlets^2^	0.06 (0.13)	403.780	**−** 0.01 (0.05)	403.894	**−** 0.03 (0.03)	403.315	0.00 (0.02)	403.891
Density of bars^2^	**−** 0.13 (0.19)	403.313	**−** 0.02 (0.03)	403.620	**−** 0.01 (0.02)	403.560	**−** 0.01 (0.01)	403.618
Crime rate^3^	0.18 (0.09)*	404.269	**−** 0.02 (0.02)	407.190	0.03 (0.24)	407.190	**−** 0.13 (0.37)	407.089
Protective factors								
Walkability^1^	**−** 0.04 (0.08)	401.915	**−** 0.02 (0.09)	402.165	0.02 (0.10)	402.145	**−** 0.03 (0.10)	402.123
NDVI^2^	0.35 (1.98)	403.883	0.68 (2.13)	403.817	1.35 (2.15)	403.571	0.57 (2.12)	403.855
Density of green spaces^2^	**−** 0.04 (0.15)	403.830	0.01 (0.07)	403.904	0.04 (0.04)	402.625	0.02 (0.03)	403.306
Density of community organizations^2^	0.07 (0.21)	403.813	**−** 0.18 (0.13)	401.684	**−** 0.02 (0.08)	403.879	0.01 (0.06)	403.898

^1^Models were adjusted for individual-level covariates and sociodemographic characteristics at the neighborhood-level. The scale used for each neighborhood-level covariates varies by outcomes (lowest AIC)

^2^Models were adjusted for individual-level covariates and sociodemographic characteristics and walkability at the neighborhood-level. The scale used for each neighborhood-level covariates varies by outcomes (lowest AIC)

^3^Models were adjusted for individual-level covariates, and sociodemographic characteristics, walkability, density of green spaces, density of off-premises alcohol outlets and density of bars at the neighborhood-level. The scale used for each neighborhood-level covariates varies by outcomes (lowest AIC)

^***^p < 0.001; **p < 0.01; *p < 0.05; †*p* < 0.10

Our results suggest an effect of the density of off-premises alcohol outlets and crime rate on DV. There was a positive association between the density of off-premises alcohol outlets within a 500 m radius and psychological DV perpetration (ß = 0.08; SE = 0.04; *p* = 0.049). Crime rate was positively associated with physical/sexual DV perpetration with radii of 500 m (ß = 0.97; SE = 0.28; *p* < 0.001) and 750 m (ß = 0.84; SE = 0.37; *p* = 0.024). The AIC of the model using a 500 m buffer was considerably smaller than that of the model using a 750 m radius (ΔAIC = 3.931). There was also an association between crime rate measured with a 250 m radius buffer and physical/sexual DV victimization (ß = 0.18; SE = 0.09; *p* = 0.050). No significant associations were identified between the density of bars and DV.

The analysis of possible protective factors revealed only a negative association between NDVI within a 1000 m radius and psychological DV victimization (ß = − 4.24; SE = 2.09; *p* = 0.044).

### Models of associations between neighborhood characteristics on DV among girls

Results from logistic regression assessing the associations between neighborhood characteristics and DV among girls are shown in Table 3.

**Table 3:** Associations between neighborhood characteristics and DV among girls

	250 m		500 m		750 m		1000 m	
	ß (SE)	AIC	ß (SE)	AIC	ß (SE)	AIC	ß (SE)	AIC
**Psychological DV perpetration**								
Risk factors								
Density of off-premises alcohol outlets^2^	0.03 (0.15)	494.899	**−** 0.01 (0.06)	494.933	**−** 0.01 (0.03)	494.892	**−** 0.01 (0.02)	494.419
Density of bars^2^	**−** 0.13 (0.14)	494.127	**−** 0.02 (0.05)	494.763	**−** 0.02 (0.02)	494.128	**−** 0.01 (0.01)	494.645
Crime rate^3^	**−** 0.13 (0.13)	497.286	**−** 0.34 (0.25)	496.838	**−** 0.73 (0.40)†	495.455	**−** 0.71 (0.48)	495.676
Protective factors								
Walkability^1^	**−** 0.10 (0.07)	496.008	**−** 0.19 (0.07)**	492.949	**−** 0.20 (0.07)**	494.037	**−** 0.19 (0.07)*	494.413
NDVI^2^	0.02 (1.56)	494.949	**−** 0.67 (1.75)	494.820	**−** 0.29 (1.90)	494.926	**−** 0.09 (1.95)	494.946
Density of green spaces^2^	0.18 (0.14)	493.110	**−** 0.02 (0.05)	494.860	0.01 (0.03)	494.827	0.01 (0.02)	494.507
Density of community organizations^2^	0.12 (0.22)	494.482	**−** 0.14 (0.16)	493.350	**−** 0.05('**−** 0.09)	494.567	**−** 0.04 (0.06)	494.276
**Physical or sexual DV perpetration**								
Risk factors								
Density of off-premises alcohol outlets^2^	0.16 (0.14)	457.140	0.00 (0.07)	458.632	0.04 (0.03)	457.265	0.01 (0.02)	458.404
Density of bars^2^	**−** 0.09 (0.15)	458.269	0.05 (0.06)	457.821	0.00 (0.02)	458.592	**−** 0.01 (0.01)	458.261
Crime rate^3^	**−** 0.00 (0.05)	460.683	**−** 0.20 (0.25)	459.964	**−** 0.66 (0.41)	456.010	**−** 0.61 (0.44)	458.209
Protective factors								
Walkability^1^	**−** 0.00 (0.07)	456.774	**−** 0.05 (0.07)	456.281	**−** 0.06 (0.07)	456.028	**−** 0.05 (0.07)	456.243
NDVI^2^	1.47 (1.66)	457.857	3.09 (1.91)	456.057	3.52 (1.99)†	455.581	3.42 (1.93)†	455.795
Density of green spaces^2^	0.03 (0.13)	458.585	0.06 (0.06)	457.536	0.03 (0.03)	457.622	0.02 (0.02)	457.867
Density of community organizations^2^	0.02 (0.21)	458.629	**−** 0.09 (0.13)	458.027	**−** 0.08 (0.08)	457.717	**−** 0.05 (0.06)	457.903
**Psychological DV victimization**								
Risk factors								
Density of off-premises alcohol outlets^2^	0.04 (0.12)	582.214	0.01 (0.05)	582.304	**−** 0.02 (0.03)	581.712	**−** 0.02 (0.02)	580.185
Density of bars^2^	**−** 0.29 (0.15)*	578.118	**−** 0.08 (0.06)	580.316	**−** 0.04 (0.02)†	579.715	**−** 0.03 (0.01)*	578.322
Crime rate^3^	**−** 0.08 (0.10)	584.017	**−** 0.14 (0.16)	584.536	**−** 0.11 (0.32)	585.194	**−** 0.28 (0.37)	584.684
Protective factors								
Walkability^1^	**−** 0.06 (0.06)	585.322	**−** 0.13 (0.06)*	582.121	**−** 0.17 (0.06)**	580.321	**−** 0.17 (0.07)*	580.406
NDVI^2^	**−** 0.01 (1.41)	582.321	**−** 0.3 (1.64)	582.287	0.36 (1.82)	582.276	0.41 (1.86)	582.265
Density of green spaces^2^	**−** 0.01 (0.13)	582.312	**−** 0.04 (0.05)	581.598	**−** 0.02 (0.03)	581.934	**−** 0.01 (0.02)	581.934
Density of community organizations^2^	0.06 (0.20)	582.208	**−** 0.07 (0.11)	581.842	**−** 0.01 (0.07)	582.315	**−** 0.04 (0.05)	581.745
**Physical or sexual DV victimization**								
Risk factors								
Density of off-premises alcohol outlets^2^	0.06 (0.12)	487.397	**−** 0.04 (0.06)	487.201	**−** 0.01 (0.03)	487.537	**−** 0.01 (0.02)	487.503
Density of bars^2^	**−** 0.10 (0.18)	487.300	**−** 0.02 (0.08)	487.494	**−** 0.01 (0.02)	487.482	**−** 0.01 (0.01)	487.317
Crime rate^3^	**−** 0.01 (0.04)	488.524	**−** 0.04 (0.15)	488.502	**−** 0.05 (0.46)	488.552	0.23 (0.50)	488.303
Protective factors								
Walkability^1^	**−** 0.15 (0.07)*	485.604	**−** 0.15 (0.07)*	486.837	**−** 0.13 (0.07)†	488.062	**−** 0.08 (0.07)	490.049
NDVI^2^	2.69 (1.56)†	484.771	3.30 (1.79)†	484.218	3.86 (1.81)*	483.195	4.25 (1.82)*	482.368
Density of green spaces^2^	0.02 (0.12)	487.574	0.06 (0.05)	486.369	0.03 (0.03)	486.950	0.03 (0.02)	485.826
Density of community organizations^2^	0.42 (0.20)*	482.699	0.13 (0.11)	486.268	0.06 (0.07)	486.763	0.02 (0.05)	487.454

^1^Models were adjusted for individual-level covariates and sociodemographic characteristics at the neighborhood-level. The scale used for each neighborhood-level covariates varies by outcomes (lowest AIC)

^2^Models were adjusted for individual-level covariates and sociodemographic characteristics and walkability at the neighborhood-level. The scale used for each neighborhood-level covariates varies by outcomes (lowest AIC)

^3^Models were adjusted for individual-level covariates, and sociodemographic characteristics, walkability, density of green spaces, density of off-premises alcohol outlets and density of bars at the neighborhood-level. The scale used for each neighborhood-level covariates varies by outcomes (lowest AIC)

^***^p < 0.001; **p < 0.01; *p < 0.05; †*p* < 0.10

Regarding risk factors, results revealed negative associations between density of bars within a buffer of 250 m (ß = − 0.29; SE = 0.15; *p* = 0.045) and 1000 m (ß = − 0.03; SE = 0.01; *p* = 0.049) and psychological DV victimization. The difference in AIC between the two models was small (ΔAIC = 0.204), suggesting that both spatial scales are comparable. No significant effects were observed for the density of off-premises alcohol outlets and crime rate.

In analyzing possible protective factors, several associations were found between walkability and DV. Walkability within a radius of 500 m (ß = − 0.19; SE = 0.07; *p* = 0.007), 750 m (ß = − 0.20; SE = 0.07; *p* = 0.007), and 1000 m (ß = − 0.19; SE = 0.07; *p* = 0.013) were negatively associated with psychological DV perpetration. The model using a 500 m radius buffer had the smallest AIC (AIC = 496.119), but the difference with the other models was negligible (ΔAIC < 1.464). There were associations between walkability in a buffer of 500 m (ß = − 0.13; SE = 0.06; *p* = 0.028), 750 m (ß = − 0.17; SE = 0.06; *p* = 0.008), and 1000 m (ß = − 0.17; SE = 0.07; *p* = 0.012) with psychological DV victimization. The model using a 750 m radius had the lowest AIC (AIC = 580.321) but was comparable with the other two models (ΔAIC < 1.800). Finally, a decrease of walkability within a radius of 250 m (ß = − 0.15; SE = 0.07; *p* = 0.029), and 500 m (ß = − 0.15; SE = 0.07; *p* = 0.049) were associated with lower physical/sexual DV victimization. The difference between AIC is small (ΔAIC = 1.233), suggesting that the two models are comparable. Significant positive associations were also observed for NDVI and density of community organizations. NDVI in a buffer of 750 m (ß = 3.86; SE = 1.81; *p* = 0.035) and 1000 m (ß = 4.25; SE = 1.82; *p* = 0.021) were related to greater physical/sexual DV victimization. Model using a 1000 m buffer had the lowest AIC (AIC = 482.368) but the difference in AIC between the two models were negligible (ΔAIC = 0.827). An increase in density of community organizations within a 250 m radius (ß = 0.42; SE = 0.20; *p* = 0.039) was also associated with greater physical/sexual DV victimization. No significant associations were found between the density of green spaces and DV.

### Correlations between buffers for neighborhood-level variables

Table 4 shows the Spearman’s rho correlation coefficients between the four buffers for each neighborhood-level variable. All correlations were significant (*p* < 0.001) with rho coefficients ranging from 0.38 to 99. There were strong correlations across buffers for walkability (from 0.82 to 0.98) and NDVI (from 0.86 to 0.99). Moderate to strong correlations were observed for crime rate (from 0.56 to 0.94), density of green spaces (from 0.47 to 0.93), density of alcohol outlets (from 0.63 to 0.96), and density of bars (from 0.43 to 0.89). Finally, there were weak to strong correlations between buffers for density of community organizations (from 0.38 to 0.86).

**Table 4:** Correlations between buffers for neighborhood-level variables

	500 m	750 m	1000 m
	Rho	Rho	Rho
250 m			
Density of off-premises alcohol outlets	0.72	0.66	0.63
Density of bars	0.60	0.48	0.43
Crime rate	0.71	0.61	0.56
Walkability	0.90	0.85	0.82
NDVI	0.95	0.89	0.86
Density of green spaces	0.61	0.57	0.47
Density of community organizations	0.63	0.47	0.38
500 m			
Density of off-premises alcohol outlets		0.90	0.85
Density of bars		0.80	0.71
Crime rate		0.88	0.81
Walkability		0.96	0.93
NDVI		0.97	0.94
Density of green spaces		0.86	0.78
Density of community organizations		0.77	0.65
750 m			
Density of off-premises alcohol outlets			0.96
Density of bars			0.89
Crime rate			0.94
Walkability			0.98
NDVI			0.99
Density of green spaces			0.93
Density of community organizations			0.86

All p values < 0.001

## Discussion

The current study aimed to contribute to the body of research on the determinants of DV by analyzing the effects of several neighborhood characteristics (criminality, greenness, walkability, density of green spaces, alcohol outlets, and community organizations) using a multi-scalar approach. To our knowledge, no other study had simultaneously assessed various forms of DV victimization and perpetration in relation to multiple neighborhood risk and protective factors. Results of this study suggest that several neighborhood characteristics could influence DV. These factors would be active at different scales, and their effects would be modified by gender with a varying amplitude depending on the form of violence considered.

Our results showed that associations between neighborhood factors and DV varied across buffers. In addition to these findings, correlations between the buffers of 500 m, 750 m, and 1000 m were strong for most variables, but correlations between the buffer of 250 m and the other buffers were weaker, suggesting that processes acting at different scales could be captured. Criminality, density of green spaces, alcohol outlets, and community organizations were specifically sensitive to the influence of scale. The sensitivity to scale in our findings is indicative that not all neighborhood determinants of DV act at the same scale. Reflecting on the choice of an appropriate scale to analyze associations between neighborhood factors and DV is essential to reduce the risk of bias in estimating the risks associated with these factors.

### Neighborhood risk factors and DV

The effects of neighborhood risk factors on DV were mainly observed for DV perpetration among boys. There was an association between crime rate within a buffer of 500 m and 750 m and the perpetration of physical/sexual violence for boys only. Although there was a strong correlation for crime rate between the buffer of 500 m and 750 m, the effect of this characteristic was stronger in the model using a buffer of 500 m (substantially lower AIC). A positive association was also observed among boys between the crime rate within a 250 m radius and physical/sexual DV victimization. To date, only studies that have used measures of perceived neighborhood disorder have found significant associations with DV [21–24]. However, perceptual measures are subject to same-source bias [79]. This bias occurs when outcomes and neighborhood characteristics are self-reported and can be interrelated. In our case, having experienced DV could influence the perception of neighborhood disorder and vice versa. Studies using police data to describe the effect of crime have found no significant association with DV [25, 26]. However, crime rate was measured at the census tract level, which corresponds to the average size of population and area of a 750 m buffer. Our results suggest that this variable may act more locally (250 m and 500 m). Exposure to neighborhood crime could lead to the normalization of violence, which may influence the adoption of violent behavior in intimate relationships [9, 80]. These processes could be mainly local. Crime tends to be concentrated in some microenvironments (e.g., blocks, street segments) [81] and adolescents living in these microenvironments could be more impacted by the negative effects of criminality. Let us note that the correlation between the buffers of 500 m and 750 m was strong but, a weaker correlation was found between the buffers of 500 m and 1000 m. In addition, crime rate in a 250 m buffer was only moderately correlated with that in the other buffers. Smaller buffers (especially the 250 m buffer) may better capture the concentration of criminality, while larger buffers could more adequately describe processes occurring at larger scales. In addition to these differences in the spatial scale of analysis, adjusting for the level of greenness, walkability and density of bars, off-premises alcohol outlets and green spaces resulted in a suppression effect. Sensitivity analyses in which all these variables were removed found no association between crime rate and physical/sexual DV perpetration and victimization (results available upon request). Suppression occurs when the inclusion of one or more variables in a model increases the effect of another variable by removing irrelevant variability in the predictors, leading to a more robust estimate of the effect [82]. The adjustment for a set of neighborhood-level variables in our models allowed to isolate the effect of crime that was not related to the physical characteristics of the environment. These results highlight the relevance of our DAG to investigate the links between crime and DV. Therefore, further research should consider contextual factors when analyzing these associations.

Access to alcohol outlets could also be a potential risk factor for boys. The density of off-premises alcohol outlets within a 500 m radius was positively associated with psychological DV perpetration among boys. Several studies exploring the links between the density of alcohol outlets and intimate partner violence have also identified significant associations [27–29]. Selling alcohol to minors is prohibited, which may make it difficult for adolescents to visit bars. However, off-premises alcohol outlets could be used to obtain alcohol through older relatives (e.g., friends, brothers, sisters) [83]. The density of off-premises alcohol outlets may thus influence consumption among adolescents [32], which may relate to an increased risk of perpetrating DV [7]. According to our results, correlations for density of off-premises alcohol outlets between the buffer of 500 m, 750 m, and 1000 m were strong, but the effect of this characteristic on DV is only observed for the 500 m buffer, suggesting that the density of these retails in the immediate neighborhood may have a more significant influence on consumption. Finally, the effect of the density of off-premises alcohol outlets was only observed for boys. This gender sensitivity may be explained by lower parental monitoring for boys than girls [55]. Furthermore, our results suggested a protective effect of the density of bars (250 m and 1000 m) on psychological DV victimization among girls. It is possible that the density of bars was confounded by the density of other services that have not been considered in this study. In particular, cafés and malls could contribute to social interaction and social cohesion in neighborhoods [84], a potential protective factor for DV [42, 43]. Moreover, let us note that the correlation between the 250 m and 1000 m buffers for density of bars was moderate (rho = 0.43), suggesting that different processes could have been captured at each scale. The 1000 m buffer could reflect the density and diversity in the neighborhoods, while the 250 m buffer could better describe the immediate proximity to services. In some residential areas where services may be highly concentrated (e.g., on a few streets), the 250 m buffer zone may better reflect the proximity of these places, which would be masked by the use of larger buffers.

### Neighborhood protective factors and DV

Among the protective factors analyzed in this study, walkability was found to be linked to several forms of DV for girls but not for boys. Increased walkability within a buffer of 500 m, 750 m, and 1000 m around participants' homes was negatively associated with psychological DV perpetration and victimization. An increase of walkability in a 250 m and 500 m buffer could also reduce the risk of physical/sexual DV victimization. As our theoretical framework suggests, this physical characteristic of neighborhoods may encourage active behavior and physical activity [49, 71, 72] as well as contribute to greater social interactions and a sense of belonging [11, 50], which could positively affect adolescent behaviors, such as reducing violent behavior [52]. These conditions could promote the development of social ties and the presence of witnesses who could act to prevent such behavior or intervene. Girls, in particular, would benefit from the positive effects of walkability. The effect of this characteristic of the neighborhoods on psychological violence was observed up to 1000 m, an acceptable walking distance for adolescents [85]. The small difference between the buffer zones in the estimates of the effect of walkability and models’ fit suggests a small influence of the spatial scale of analysis for this variable. This minor scaling effect was also observed when analyzing correlations between buffers. All buffers were strongly correlated (rho ranging from 0.82 to 0.98). However, walkability could have a more local influence (250 m and 500 m) for the more severe forms of DV (physical/sexual). Walkability could foster collective efficacy [11], which may depend on social interactions and proximity between residents, as other social processes [86]. Walkability of the local environment could affect these social processes, while walkability on a larger scale could have a greater influence on active behavior. Future studies should investigate these potential mediation effects to provide a better understanding of the mechanisms through which walkability acts on DV.

The density of community organizations in a buffer of 250 m was positively associated with physical/sexual DV victimization among girls. This result seems counter-intuitive as these resources tend to positively affect adolescents by offering them supervised activities and opportunities to develop their social ties [10, 44]. However, some studies suggest that organizations with little structured activities may have deleterious effects on adolescents by promoting interactions between at-risk individuals [87]. The presence of these services in the immediate environment of girls may increase their risk of physical or sexual victimization due to a greater presence of adolescents adhering to norms and attitudes that encourage violence. To test this hypothesis, future studies should identify the effect of community organizations by distinguishing between the different types of organizations and considering the services offered by these organizations. These studies should also consider the influence of the spatial scale of analysis. The effect of density of community organizations was found only for the buffer of 250 m. In addition, correlations between the 250 m buffer and the other buffers ranged from moderate to weak. The scale sensitivity for this variable could be explained by the small number of community organizations (n = 423) and suggest the importance of choosing the scale of analysis carefully. Finally, let us note that no effect was observed for boys and other forms of violence for girls. The availability of these resources is not always associated with their use by adolescents, which may depend on other conditions such as the interest of adolescents and the cost of the activities [88]. Therefore, it would be relevant to further analyze the effect of adolescents' use of community resources using measures of social participation in order to adequately assess the effect of these environments.

Associations between greenness and DV were also observed but, the direction of this relationship varies by gender. Greenness in the 1000 m radius buffer could have a protective effect on psychological DV victimization among boys. Conversely, an increase in greenness within a buffer of 750 m and 1000 m was associated with a greater risk of physical/sexual DV victimization among girls. Although no study has evaluated the effect of greenness on DV, this feature of neighborhoods could reduce aggressive behavior [37], a potential risk factor for DV victimization [89]. Our results showed that boys are more sensitive to the protective effects of greenness than girls. For girls, higher greenness would have a deleterious effect on DV victimization, suggesting that this link could involve other variables that were not measured, such as parental supervision and social control. The characteristics of the physical environment may influence parents' perception of the neighborhood, and positive attributes could lead to a more permissive parenting style regarding outdoor activities [90]. In neighborhoods with a high level of greenness, girls may have more freedom to go out alone, without parental supervision. However, such neighborhoods could be associated with the presence of shielded areas with a limited presence of adults resulting in opportunities for unsupervised activities [91]. Such contexts could, in turn, increase the risk of DV victimization for girls. Furthermore, correlations between buffers showed a minor scaling effect for greenness (rho ranging from 0.86 to 0.99). The variation in the level of greenness could be mainly explained at larger scales, as there would be little local variation in this factor. The buffer of 750 m and 1000 m, for which the effects of greenness were observed, may better capture these processes. These results are consistent with those reported by Younan et al. [37] in that the effects of greenness are observed at larger scales only (1000 m for boys and 750 m and 1000 m for girls). These radii could correspond to the areas used by adolescents. The 1000 m distance corresponds to about 15 min of walking and is consistent with the travel distance by walking of adolescents [85].

Finally, no association between the density of green spaces and any form of DV was found. The density of these resources is not necessarily associated with greater use. Some characteristics of green spaces, such as facilities (e.g., sports fields, picnic tables), could be more predictive of adolescents’ use [92]. Further studies should examine the effect of access to green spaces on DV, considering their characteristics. The influence of the spatial scale of analysis should also be considered because a potential scaling effect for the density of green spaces may exist. Our results showed that correlations between the buffer of 500 m, 750 m, and 1000 m for the density of green spaces were strong. However, these buffers were only moderately correlated with the buffer of 250 m. The 250 m buffer may better capture proximity to this resource, while larger buffers would be more representative of density within neighborhoods.

### Strengths and limitations

This study contributes to the research on neighborhoods’ factors of DV and demonstrates the importance of considering scale when analyzing these associations. The effects of some neighborhood characteristics related to social disorganization theory that had already been studied in the United States were analyzed, as well as other characteristics of the physical environment that had not yet been documented. This study also provides a multi-scalar understanding of neighborhood-level processes and highlights the importance of neighborhood definition.

Some limitations should be considered. Firstly, self-reported data for DV are at risk of social desirability bias. This bias could not be controlled due to the absence of a social desirability measure in the QHSHSS. Secondly, physical and sexual DV were assessed jointly as the prevalence of sexual DV perpetration for girls was low (2%). Strong correlations between these forms of violence for both girls and boys were observed in our data, suggesting that physical and sexual DV may co-occur. Using such a measure is also consistent with other studies reporting on the relationship between DV and neighborhood factors [25–29]. However, measuring physical and sexual DV jointly did not make it possible to identify factors specifically associated with sexual DV. Low-prevalence outcomes could be more sensitive to outliers and require a large sample to ensure a representative sample for analyzing the influence of neighborhood-level factors. Future studies on the relationship between neighborhoods’ characteristics and sexual DV should therefore focus on sexual DV. Thirdly, more than 26% of the participants were excluded because they did not provide a valid postal code. This information was required to assign to respondents the neighborhood-level variables. Missing data on postal codes led to a reduction in the sample size, which reduced statistical power. However, let us note that these missing data should not substantially impact our estimates as they are randomly distributed for all covariates, except for grade level among girls. Fourthly, social environment characteristics were not analyzed in this study but could influence DV. Some studies have focused on the associations between DV and collective efficacy [23, 43, 53, 54], a concept related to social disorganization theory [17]. However, collective efficacy was not assessed in the QHSHSS, and the spatial density of respondents was too low to derive neighborhood-level variables from individual responses. Studies in urban areas most often used measurements at the individual level [23, 43, 53, 54], which are limited in neighborhood-level processes. Rothman et al. [43] measured collective efficacy at the neighborhood level, but neighborhoods were operationalized with larger spatial units than the census tracts*.* To our knowledge, no study has assessed the effect of collective efficacy using small spatial units as a proxy of the local environment. Therefore, future research should develop methods to estimate the characteristics of the social environment at fine scales and further investigate the influence of these factors on DV by considering scale effects. Small area estimation methods [93] are promising tools for estimating components of the social environment in small spatial units from survey data. Lastly, logistic regressions performed in this study did not consider the spatial dependency of the data. However, spatial pattern in neighborhoods’ characteristics as well as health outcomes have been observed in previous research. For example, a study found a spatial patterning of neighborhood physical characteristics and depressive symptoms among adolescents [94]. Authors suggested that depressive symptoms could be clustered due to social interactions (e.g., neighborhood peer effects) and exposure and response to the same neighborhood factors. Similar processes could also be observed with DV. Future studies evaluating associations between neighborhood characteristics and DV should assess the potential spatial pattern of DV by considering the spatial dependency of data. Spatial regressions would address this issue by taking into account spatial autocorrelation (i.e., level of similarity/dissimilarity between neighboring observations) and spatial non-stationarity (i.e., spatial variability in the amplitude and direction of estimated effects) [95]. However, these methods require an adequate spatial density of participants, which is rarely the case for national surveys. Future studies should therefore conduct large sample surveys to address this objective.

### Conclusions

Multiple neighborhood characteristics could influence DV at different scales. Social disorganization theory, often referred to for explaining these relationships [9], could only partially explain the neighborhood effects. Our results suggest that some physical characteristics of neighborhoods, such as walkability, could have a protective effect on DV. Future studies should investigate the effect of the physical environment on these behaviors by analyzing underlying processes (e.g., social support, social participation). Furthermore, our study showed that there is no single scale to assess the effect of neighborhoods’ characteristics. Therefore, the comparison of scales should be systematic to consider the multiscale effects of neighborhoods’ characteristics.

Our results regarding the association between neighborhood characteristics and DV could be observed in several geographic contexts but may not be applicable to urban contexts of the Global South. In effect, studies on neighborhoods’ effects on DV conducted outside of the United States and Canada are still rare. Specifically, cities in the Global South are different from those in North America (e.g., presence of informal settlements, larger socioeconomic and health inequalities, and extreme urban growth). Such differences may cause the neighborhood-level factors analyzed in our study to influence DV differently in the Global South, namely, if other neighborhood-level factors contribute to the incidence of DV. Despite these issues, neighborhood effects have been observed in several countries. For example, empirical studies found associations between some neighborhood factors, such as sociodemographic characteristics, neighborhood disorder and crime, and intimate partner violence among adults in Europe [96], Asia [97] as well as Africa [98]. Associations between neighborhoods’ characteristics and DV among adolescents may therefore be observed in different regions of the world, but local specificities could lead to differences in the effects of the factors analyzed in this study.

Although the relationship between neighborhood factors and DV may depend on geographic context, the influence of spatial scale of analysis is likely to affect most types of local environments, suggesting that our results on scale sensitivity are not limited to the North American context. In effect, the neighborhood factors analyzed in the current studies are likely to influence individuals in different regions of the world. Several studies around the world have suggested neighborhood effects on health outcomes, and some have shown potential scale effects for certain factors. For example, a literature review reported that greenness and access to green spaces could influence health outcomes in different regions of the world, including the Global South, and suggested that these effects could depend on the spatial scale of analysis (e.g., large versus small buffers to operationalize exposure to green space) [99]. Furthermore, the egocentric neighborhood is a promising approach that could be easily implemented in various geographic settings. Although the definition of administrative units (size and shape) varies across countries, egocentric neighborhoods ensure consistency in operationalizing neighborhoods, which may increase the comparability of studies. Finally, measures used in the current study to operationalize neighborhood factors are easily replicable, and some have been validated in different contexts. For example, assessing greenness by using NDVI is consistent with several studies around the world [99]. The walkability index has also been used in many countries in the Global North [71–73, 100] as well as in the Global South [100].

This study also has practice implications. Our results highlight the importance of physical features of neighborhoods that are modifiable and reach large numbers of people [101]. Urban planning and public policies could represent new avenues in efforts to efficiently prevent DV. Interventions implemented around the world to improve the physical local environment (e.g., greening, increasing density of facilities, streetscape improvements) showed positive effects on adolescent behaviors and health outcomes [102, 103]. Improving the physical environment would also promote social cohesion and residents' engagement in community life, which could benefit individuals [104]. In the United States, an intervention that aimed at greening neighborhoods and improving social cohesion through residents’ engagement has shown promising results in preventing violence and crime [105]. Such programs could be effective in reducing DV and could also have positive effects on adolescents’ health and well-being.

## Supplementary Information

**Additional file 1:** Directed Acyclic Graph.

## Data Availability

Data from the Québec Health Survey of High School Students are provided by Québec Statistics Institute and are not publicly available. Statistics Canada 2016 Census data at the dissemination area level are publicly available: https://www150.statcan.gc.ca/n1/en/catalogue/98-401-X2016044. Data on the population size of the dissemination blocks are also provided by Statistics Canada and are publicly available: https://www150.statcan.gc.ca/n1/en/catalogue/92-163-X. All other data used and analyzed during the current study are available from the corresponding author on reasonable request.
