# Supplementary material for: Associations between neighborhood characteristics and dating violence: does spatial scale matter?
Source: Int J Health Geogr. 2022 Jun 20;21:6. doi: 10.1186/s12942-022-00306-3 (PMC9210619; doi:10.1186/s12942-022-00306-3)
Supplement: Supplementary file 1 — Additional file 1: Directed Acyclic Graph. [file 12942_2022_306_MOESM1_ESM.docx]

# Directed acyclic graph (DAG)

A Directed acyclic Graph (DAG) is a graphical representation of causal assumptions. In DAG, nodes (i.e., variables) are connected by directed edges (i.e., arrows) to form a graph. A sequence of connected nodes is a path. According to the DAG theory, confounding bias can occur when there are unblocked (or open) backdoor paths. A backdoor path may be observed when the exposure and outcome share a common cause. Conditioning for this factor (e.g., adjustment) closes the path, thus avoiding the risk of confounding. Therefore, a DAG allows to determine the minimal set of adjustments in a model by identifying confounding variables. Similarly, it allows for the identification of colliders, thus limiting collider bias. For more details, see [1].

Figure 1 shows the DAG used in the current study. This DAG was created and analyzed with the web tool DAGitty (<http://www.dagitty.net/>) [2]

A1 Directed acyclic graph


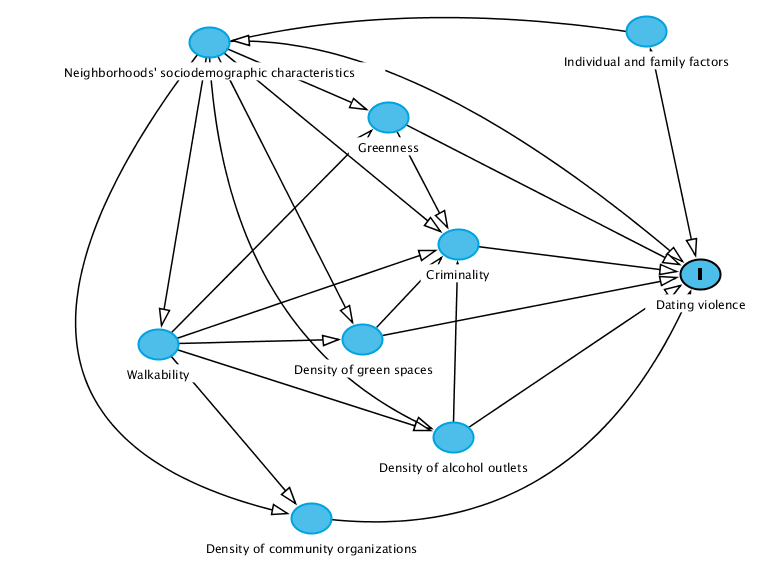


# Bibliography

1. VanderWeele TJ, Robins JM: **Directed Acyclic Graphs, Sufficient Causes, and the Properties of Conditioning on a Common Effect**. *Am J Epidemiol* 2007, **166**(9):1096-1104.

2. Textor J, Hardt J, Knüppel S: **DAGitty: a graphical tool for analyzing causal diagrams**. *Epidemiology* 2011, **22**(5):745.
